# Intestinal CD11b^+^ B Cells Ameliorate Colitis by Secreting Immunoglobulin A

**DOI:** 10.3389/fimmu.2021.697725

**Published:** 2021-11-03

**Authors:** Ying Fu, Zhiming Wang, Baichao Yu, Yuli Lin, Enyu Huang, Ronghua Liu, Chujun Zhao, Mingfang Lu, Wei Xu, Hongchun Liu, Yongzhong Liu, Luman Wang, Yiwei Chu

**Affiliations:** ^1^ Department of Immunology, School of Basic Medical Sciences, and Institutes of Biomedical Sciences, Fudan University, Shanghai, China; ^2^ Department of Pathology, The University of Hong Kong, Hong Kong, Hong Kong, SAR China; ^3^ Department of Biomedical Engineering, Columbia University, New York, NY, United States; ^4^ Department of Gastroenterology, Zhongshan Hospital, Fudan University, Shanghai, China; ^5^ State Key Laboratory of Oncogenes and Related Genes, Shanghai Cancer Institute, Renji Hospital, Shanghai Jiaotong University, School of Medicine, Shanghai, China; ^6^ Department of Endocrinology and Metabolism, Shanghai Fifth People’s Hospital, Fudan University, Shanghai, China; ^7^ Biotherapy Research Center, Fudan University, Shanghai, China

**Keywords:** CD11b^+^ B cells, IgA, DSS-induced colitis, TGF-β, Smad

## Abstract

The intestinal mucosal immune environment requires multiple immune cells to maintain homeostasis. Although intestinal B cells are among the most important immune cells, little is known about the mechanism that they employ to regulate immune homeostasis. In this study, we found that CD11b^+^ B cells significantly accumulated in the gut lamina propria and Peyer’s patches in dextran sulfate sodium-induced colitis mouse models and patients with ulcerative colitis. Adoptive transfer of CD11b^+^ B cells, but not CD11b^−/−^ B cells, effectively ameliorated colitis and exhibited therapeutic effects. Furthermore, CD11b^+^ B cells were found to produce higher levels of IgA than CD11b^−^ B cells. CD11b deficiency in B cells dampened IgA production, resulting in the loss of their ability to ameliorate colitis. Mechanistically, CD11b^+^ B cells expressed abundant TGF-β and TGF-β receptor II, as well as highly activate phosphorylated Smad2/3 signaling pathway, consequently promoting the class switch to IgA. Collectively, our findings demonstrate that CD11b^+^ B cells are essential intestinal suppressive immune cells and the primary source of intestinal IgA, which plays an indispensable role in maintaining intestinal homeostasis.

## Introduction

The intestine is a relatively independent regional immune organ, with bacteria, epithelial cells, and immune cells interacting to maintain homeostasis (1). Gut-associated lymphoid tissue (GALT) is an important site for resident immune cells to function as both a regulator and an effector (2). GALT cells, such as dendritic cells, macrophages, T cells, and B cells are widely known to be involved in maintaining a stable state of the intestinal tract (3, 4). Among the substances produced by immune cells, IgA is considered an essential factor in immune hemostasis (5–7). However, no study has reported whether there are specific subsets of B cells that produce substantial IgA in the inflammatory environment and the underlying mechanisms.

CD11b, a protein subunit that forms the integrin, alpha-M beta-2 molecule (Mac-1), with CD18, is usually expressed on myeloid cells such as macrophages and dendritic cells. CD11b has been functionally implicated in leukocyte suppression, migration, and adhesion (8–10). For instance, CD11b activation promotes pro-inflammatory macrophage polarization and functions as a negative regulator of immune suppression as well as a target for cancer immune therapy (11). In lymphocytes, CD11b is expressed on peritoneal cavity B1 B cells with unrevealed functions (12), and also expressed on some age-associated B cells (13, 14). Kunisawa et al. demonstrated that CD11b is essential for IgA^+^ plasma cell production, which is dependent on the Peyer’s patch (PP) lymphoid structure, IL-10 and MyD88 pathway (15). Our previous work revealed that CD11b is also expressed on classical B2 B cells, and CD11b^+^ B cells are essential for maintaining immune tolerance in the liver (16). Thus, whether CD11b^+^ B cells exist in GALT and whether the expression of CD11b in intestinal B cells determines the function of B cells in the maintenance of intestinal homeostasis are important questions that should be addressed.

In this study, we found that CD11b^+^ B cells accumulated in GALT under intestinal inflammatory conditions in a dextran sulfate sodium (DSS)-induced colitis model and human ulcerative colitis (UC) patients. Importantly, adoptive transfer (AT) of Peyer’s patches (PP)-derived wild-type (WT) B cells, but not *Itgam^−/−^* (CD11b KO) B cells, inhibited colitis in *Cd79a^−/−^* B cell-deficient mice, suggesting that CD11b is indispensable for the regulatory function of GALT B cells. Furthermore, compared to CD11b^−/−^ B cells, CD11b^+^ B cells produced higher levels of IgA, and exhibited more intense activation of the TGF-β signaling pathways that is associated with IgA class switching, including higher expression of TGF-β receptor and increased phosphorylation of Smad2/3. In summary, the findings of this study demonstrate that, owing to their high activity and sensitivity to the microenvironment, intestinal CD11b^+^ B cells are the major source of IgA production, with therapeutic potential for intestinal diseases, such as colitis.

## Materials and Methods

### Mice

Wild type (WT) C57BL/6 mice were obtained from the Shanghai SLAC Laboratory Animal Co., Ltd. (Shanghai, China). B6.129S4-Itgam^tm1Myd^/J (*Itgam^−/−^*) mice were kindly provided by Prof. Xiao Su of the Institute Pasteur of the Chinese Academy of Science (Shanghai, China). C57BL/6-Cd79a^tm1cyagen^ (*Cd79a^−/−^*) and C57BL/6-iga^em1cyagen^ (*Iga^−/−^*) mice were obtained from Cyagen Animal Co., Ltd. (Shanghai, China). B6.129P2-Il10tm1Cgn/J (*Il10^−/−^*) mice were obtained from The Jackson Laboratory (Bar Harbor, ME, USA). The B6.C(Cg)-Cd79a^tm1(cre)Reth^/EhobJ (*Mb-1^cre^*) mice were kindly provided by Zhongjun Dong of the School of Medicine, Tsinghua University (Beijing, China). The Tgfbr2^flox/flox^ (*Tgfbr2^f/f^*) mice were obtained from the NCI Mouse Repository (NCI-Frederick, NIH, USA) and kept in B6 background. All mice were 6–12 weeks old and were bred and housed in the animal facility at Fudan University (Shanghai, China) under specific pathogen-free barrier conditions (temperature: 18–29°C; humidity: 40%–70%; air velocity: <0.18 m/s; air pressure: 20–50 Pa). All mice were studied following protocols approved by the Ethics Committee of Animal Care and Use, Department of Laboratory Animal Science, Fudan University (Shanghai, China; approval no. 201901002Z). All animal experiments complied with the National Institute of Health’s Guide for the Care and Use of Laboratory Animals (NIH Publications No. 8023, revised in 1978).

### DSS-Induced Colitis

The experimental colitis model was established as described previously (17). Briefly, 2.5% DSS (w/v; MP Biomedicals, Santa Ana, CA, USA) was added to the drinking water administered to mice for 7 days; thereafter, the animals were administered normal drinking water. During the study period, the mice were monitored daily to conduct clinical evaluations, such as their body weight, stool consistency, and gross bleeding. Clinical assessment of disease activity was performed using the following validated scoring system: body weight loss (scored as: 0, none; 1, 1%–5%; 2, 6%–10%; 3, 11%–15%; 4, >15%), stool consistency (scored as: 0, normal pellets; 2, loose stools; 4, diarrhea), and fecal blood (scored as: 0, negative hemoccult test; 1, positive hemoccult test; 2, blood visibly present in the stool; 3, blood visibly and blood clotting on the anus; 4, gross bleeding) (18). The disease activity index (DAI) was calculated using the above parameters (19–21).

### Tissue Histopathology

On different days after DSS induction, mice were sacrificed, and their colon tissues were removed and fixed in 10% buffered formalin. Paraffin sections (6 μm) of the distal segments of the colon were cut using a manual rotary microtome (Leica RM2235, Wetzlar, Germany) and stained with hematoxylin and eosin. Histological assessment of colon injury was mainly based on the infiltration of inflammatory cells and epithelial damage and was scored from 0 to 4 as follows: 0, normal tissue; 1, inflammation with scattered infiltrating inflammatory cells and no signs of epithelial degeneration; 2, multiple foci and/or mild epithelial ulcerations; 3, severe inflammation with marked wall thickening and/or ulcerations in >30% of the tissue section; and 4, inflammation with transmural inflammatory cell infiltration and loss of the entire crypt and epithelium. The average fields of view per colon were blindly evaluated for each mouse as described previously (22, 23). The cumulative DSS histopathological scores of individual *Cd79a^−/−^* mice treated with DSS were calculated by evaluating several tissue pathology parameters.

### Flow Cytometry

Mononuclear cells were first incubated with anti-CD16/32 antibody (BD Bioscience, San Jose, CA, USA) and then reacted with the following anti-mouse antibodies: phycoerythrin (PE)-CD11b (M1/70, eBioscience, San Diego, CA, USA), allophycocyanin (APC)-eFluor780-CD11b (M1/70, eBioscience), APC-CD19 (6D5, BioLegend), APC-Cy7-CD19 (1D3, eBioscience), fluorescein isothiocyanate(FITC)-IgA (C10-3, BD Biosciences), Pacific blue-CD45 (30-F11, BioLegend), APC-IL-10 (JES5-16E3, BioLegend), PE-TGFβ receptorII (TGF-βRII, polyclonal, FAB532P, R&D Systems, Minneapolis, MN, USA), Brilliant Violet 421-TGF-β1 (TW7-16B4, BioLegend), PE-IL-6 (MP5-20F3, BioLegend), PE-Smad1 (pS463/pS465)/Smad8 (pS465/pS467) (N6-1233, BD Biosciences), PE-Smad2 (pS465/pS467)/Smad3 (pS423/pS425) (O72-670, BD Biosciences), PE-CD95 (APO-1/Fas) (15A7, eBioscience), FITC-CCR9 (CW-1.2, BioLegend), APC-α4β7 (DATK32, BioLegend), APC-CD62L (MEL-14OX85, BioLegend), FITC-CD103 (2E7, BioLegend), PE-CD69 (H1.2F3, eBioscience), APC-eFluor 780-CD44 (IM7, eBioscience), PE-CCR7 (3D12, eBioscience), PE-PD-L2 (TY25, eBioscience), PE-Cyanine7-CD21/35 (7G67E9, BioLegend), eFluor 450-CD23 (B3B4, eBioscience), APC-CD43 (1G10, eBioscience), eFluor 450-IgD (11-26c, eBioscience), PE-Cyanine7-IgM (II/41, eBioscience), Pacific Blue-CD38 (Clone 90, BioLegend), APC-CD45.1 (A20, eBioscience), FITC-CD45.2 (Clone 104, eBioscience), FITC-CD40 (HM40-3, eBioscience), APC-CD86 (GL-1, eBioscience), eFluor 450-CD80 (16-10A1, eBioscience), APC-CD138 (Clone 281-2, BioLegend), FITC-MHC II (M5/114.15.2, eBioscience), Alexa Fluor 488-PNAd (MECA-79, eBioscience), eFluor 660-GL7 (GL7, Biolegend), PE-S1P1 (Clone 713412, R&D System), APC-CCR10 (248918Polyclonal, R&D System), TGF-β (TW7-16B4A), Smad1 (pS463/pS465)/Smad8 (pS465/pS467) (N6-1233), Smad2 (pS465/pS467)/Smad3 (pS423/pS425) (O72-670), PE-Blimp-1 (5E7, eBioscience), eFluor 450-IL-6 (MP5-20F3, eBioscience), and FITC-Ki67 (SolA15, eBioscience). The human antibodies used were eFluor 450-CD19 (HIB19, eBioscience), PE-Cyanine7-CD11b (ICRF44, eBioscience), FITC-IgA (IS11-8E10, BioLegend), PE-TGF-βRII (25508, R&D Systems), PerCP-Cy5.5-TGF-β (TW4-9E7, BD Biosciences), PE-Smad2 (pS465/pS467)/Smad3 (pS423/pS425) (O72-670, BD Biosciences), APC-CCR9 (L053E8, BioLegend), and PE-α4β7 (DATK32, eBioscience).

Mononuclear cells were incubated with anti-CD16/32 antibody (eBioscience) and reacted with the Fixable Viability Dye eFluor 506(Invitrogen) and anti-mouse cell surface antibodies. To stain intranuclear molecules, cells were first fixed and permeabilized with the Staining Buffer set (eBioscience) according to the manufacturer’s instructions and then stained with anti-mouse antibodies. To stain intracellular cytokines, cells were stimulated with Cell Stimulation Cocktail (plus protein transport inhibitors) (eBioscience) for the last 5 h, followed by fixation and permeabilization with Staining Buffer set (eBioscience) according to the manufacturer’s instructions. Finally, the cells were stained with anti-mouse antibodies. For phosphorylation detection, PP-derived CD11b^+^ B cells and *Itgam^−/−^* B cells were stimulated with lipopolysaccharide (LPS, 10 µg/mL, Sigma-Aldrich, St. Louis, MO, USA), B-cell-activating factor of the tumor-necrosis-factor family (BAFF, 25 ng/mL, R&D Systems), and TGF-β (2 ng/mL, R&D Systems) for 0, 5, 15, and 30 min. Formaldehyde was added at a final concentration of 2%, and surface staining was performed using anti-CD11b and anti-CD19 antibodies. After permeabilization using phosflow perm buffer (BD Bioscience), the cells were resuspended in incubation buffer containing the antibodies against phosphorylated-Smad1/8(p-Smad1/8) and p-Smad2/3 for 30 min at room temperature and washed with additional antibodies for another 30 min. Finally, phosphorylated detection was performed using flow cytometry.

The concentrations of the matched isotype antibodies were used as the negative controls. Flow cytometric analysis was performed using a CyAn ADP Analyzer (Beckman Coulter, Miami, FL, USA), and data were analyzed with FlowJo software (Tree Star, Ashland, OR, USA). The absolute number of cell populations was calculated based on their respective percentages using flow cytometry.

### B Cell Isolation and AT

B cells (CD19^+^) from mouse PPs on day 0 were purified using B cell isolation kits from StemCell Technologies (Vancouver, BC, Canada) according to the manufacturer’s recommended protocol. CD11b^+^ B cells and CD11b^−^ B cells were sorted using a MoFlo flow cytometer (FCM; Beckman Coulter). The purity of the isolated cell population was at least 90%. To investigate the role of CD11b in B cells in colitis, approximately 1 × 10^7^ purified single-cell suspensions derived from WT or *Itgam^−/−^* mice were injected into each *Cd79a^−/−^* mouse through the inferior ophthalmic vein 48 h before DSS consumption. The control group received phosphate-buffered saline (PBS). In the assay of the cell-based therapy for colitis, purified CD11b^+^ B cells (5 × 10^6^ cells per mouse) and CD11b^−^ B cells were adoptively transferred in the same manner.

### Animal Treatment

2.5% DSS (w/v; MP Biomedicals, Irvine, CA, USA) was added to the drinking water given to the mice for 7 days followed by providing normal drinking water.

### Isolation of GALT Cells

To isolate mononuclear cells from MLNs and PPs, the cells were carefully removed with fine scissors and ground with RPMI-1640 medium (Gibco, Grand Island, NY, USA) containing 2% fetal bovine serum (FBS, GIBCO BRL, Invitrogen, Carlsbad, CA, USA). Mononuclear cells from colorectal lamina propria (LP) were isolated using the method described by Lefrancois and Lycke (24). Briefly, the colon was lavaged with RPMI-1640 to remove fecal matter and debris, opened longitudinally, cut into 1 cm pieces, and stirred twice in RPMI-1640 medium containing 10 mM HEPES, 25 mM NaHCO_3_, 1 mM DTT, 1 mM EDTA (all from Sigma-Aldrich, St. Louis, MO, USA), and 2% fetal calf serum for 30 min at 37°C. To isolate LPs, the remaining pieces were digested with RPMI-1640 medium plus 1 mg/mL collagenase (Roche Applied Science, Upper Bavaria, Germany), 40 μl/mL dispase (Sigma-Aldrich), and 4 μl/mL DNase I (Sigma-Aldrich) for 60 min at 37°C. Mononuclear cells of LPs were then purified with discontinuous Percoll (GE Healthcare, Little Chalfont, Buckinghamshire, UK) gradient centrifugation by collecting interface cells between the 40% and 70% layers.

### *In Vitro* Culture

For CD11b^+^ B cell stimulation, purified PP-derived B cells were stimulated with LPS(10μg/mL, Sigma-Aldrich), BAFF(25ng/mL, R&D Systems), and/or cytokines(all from R&D Systems). For IgA^+^ cell stimulation, CD11b^+^ B cells and *Itgam^−/−^* B cells were stimulated with LPS(10μg/mL), BAFF(25ng/mL),and TGF-β (4ng/mL) for 72 h. All purified cells were plated at 5 × 10^5^/mL in RPMI-1640 medium containing 10% FBS. LY2109761 (5 μM, Selleck, Houston, TX, USA) and anti-TGF-β (1D11.16.8) (4 μg/mL, BioXCell, West Lebanon, NH, USA) were added to some groups. Flow cytometry was performed to measure the expression of IgA isotypes in cultured B cells using PE-IgA (mA-6E1, eBioscience).

For human peripheral blood mononuclear cell (PBMC) stimulation, isolated PBMCs were stimulated with CpG (2μg/mL, ODN 2216, InvivoGen), BAFF (25ng/mL, R&D Systems), and TGF-β (2ng/mL, R&D Systems) for 72 h. All cells were plated at 5 × 10^5^/mL in RPMI-1640 medium containing 10% FBS.

### Transfection of Small Interfering RNAs

WT splenic B cells were purified using the EasySep™ MouseB Cell Isolation Kit (Stemcell Technologies, Vancouver, Canada). Isolated splenic B cells were washed three times and then transfected with TGF-β small interfering RNA (siRNA) (siTGFβ) or negative control siRNA (siNC, 100nM) by electroporation in Opti-MEM medium. The ECM830 Electro Square Wave Porator (Harvard Apparatus BTX, USA) was set to 400 volt and 700 μs electric shock for the suspension B cells. The transfected cells were cultured in corresponding stimulations (LPS 10ug/mL; BAFF 25ng/mL; LY2109761 5 µM; and anti-TGF-β 4 µg/mL). As siRNAs partially enter suspension cells by electroporation, total mRNA was extracted 24 h after the transfection to detect the transfection efficiency. Based on the significant knockdown efficiency, the RNA level of TGF-β was detected using real-time polymerase chain reaction (PCR) and the percentage of IgA^+^ B cells was measured using flow cytometry at 72 h, and the production of IgA in the supernatant was determined using enzyme-linked immunosorbent assay (ELISA).

siRNA:
siTGFβ (forward)- 5′-GAAGCGGACUACUAUGCUAdTdT-3′
siTGFβ (reverse)- 5′-UAGCAUAGUAGUCCGCUUCdTdT-3′
siNC (forward)- 5′-UUCUCCGAACGUGUCACGUdTdT-3′
siNC (reverse)- 5′-ACGUGACACGUUCGGAGAAdTdT-3′
real-time PCR primers:
β-actin (forward)-5′-CTACCTCATGAAGATCCTGACC-3′
β-actin (reverse)-5′-CACAGCTTCTCTTTGATGTCAC-3′
TGFβ (forward)-5′-CCAGATCCTGTCCAAACTAAGG-3′
TGFβ (reverse)-5′-CTCTTTAGCATAGTAGTCCGCT-3′

### Colony-Forming Units (CFU) Calculation

WT mice and *Iga^-/-^* mice were provided drinking water with 2.5% DSS (w/v; MP Biomedicals, Santa Ana, CA) for 7 days, followed by normal drinking water. The SPF faculties, sterile food, and water were strictly controlled during this process. Twenty milligrams of feces were collected every hour on day 10. The quantitative feces were diluted with 1 mL of sterile PBS. After the bacterial solution was mixed evenly, the bacterial solution was diluted 10^4^-fold, and 50 uL of the diluted bacterial solution was dropped into the solid LB medium and then evenly smeared with a laboratory coating rod. The coated culture dish was inverted and incubated overnight at 37°C for colony counting and CFU calculation.

### IgA-Specific Gut Microbiota Detection

In the DSS-induced colitis model, the mice were bled on day 10. Sera were collected for IgA-specific gut microbiota measurements.

1) ELISA plate with 100 μl/well of capture anti-IgA antibody in Coating Buffer. Seal the plate and incubate overnight at 4°C. Aspirate wells and wash twice with 400 μl/well Wash Buffer. 2) Block wells with 250 μl of Blocking Buffer. Incubate at room temperature for 2 hours. Aspirate wells and wash twice with 400 μl/well Wash Buffer. 3) 10^2^-fold-diluted sera was added incubate overnight at 4°C. Aspirate wells and wash five times with 400 μl/well Wash Buffer. 4) For fluorescence conjugation, the gut microbiota was incubated for 5 min with carboxyfluorescein succinimidyl ester (CFSE, ab113853, Abcam) and then washed with 10 ml of 1% FBS-PBS. Then, 50 ul CFSE-labeled gut microbiota was incubated in the sera-coated plate for 1h at room temperature in a biosafety cabinet. Aspirate wells and wash five times with 400 μl/well Wash Buffer. to remove the unruptured microbiota. 5) The plate was read at an excitation wavelength of 492 nm (Tecan). We used OD values to represent the abundance of IgA-specific gut microbiota.

### Immunofluorescence Analysis

For mouse colon, after deparaffinization, antigen retrieval, and blocking, the paraffin sections of colon tissues were incubated with the primary antibodies rabbit anti-mouse CD19 (ab227019, Abcam) and rat anti-mouse CD11b (ab8878, Abcam) overnight at 4°C. The tissues were then washed with PBS and incubated with the appropriate fluorophore-conjugated secondary antibodies: Alexa Flour-488 (A-21206, Invitrogen) and 647 (A48265TR, Invitrogen) at a dilution of 1:100 in 5% bovine serum albumin (BSA) for 1 h at room temperature. Additionally, 4′, 6-diamidino-2-phenylindole (DAPI) was used as a counterstain. Images were acquired using a spectral confocal microscope (TCS SP5, TCS SP8; Leica, Wetzlar, Germany).

Colon tissues were obtained from six patients. This study was approved by the Medical Ethics Council of Zhongshan Hospital (approval number: Y2016-197). Informed consent was obtained from all the patients recruited in this study. After deparaffinization, antigen retrieval, and blocking, paraffin sections of colon tissues were incubated with primary antibodies against hCD19, hCD11b, and hIgA (all from Abcam, Cambridge, MA, USA) overnight at 4°C. The tissues were then washed with PBS and incubated with the appropriate fluorophore-conjugated secondary antibodies, Alexa Flour-488, 594, and 633 (all from Invitrogen) at a dilution of 1:200 in 5% BSA for 1 h at room temperature. DAPI was used as a counterstain. Images were acquired using a spectral confocal microscope (TCS SP5, TCS SP8; Leica).

The density was calculated using Image J software (National Institutes of Health), and the procedures were as follows: For a single-channel (monochromatic) fluorescence image, the gray value of each pixel represented the fluorescence intensity at that point. The fluorescence intensity formula for a specific region was average fluorescence intensity (mean) = total fluorescence intensity of the region (IntDen)/Area of the region (Area) (Mean: Mean gray value; IntDen: Integrated Density). 1) After the image was added, single image-color-split channels were extracted. If the image was stored in RGB format, the channel needed to be divided first; if the image was 16-bit or 8-bit, the threshold operation could be performed directly. 2) The threshold was adjusted, and the appropriate area was selected (image-adjust-threshold). The software automatically selected a default value and selected the default value uniformly (to eliminate the error caused by manually selecting the threshold value for different photos, it is better to use the default threshold value). 3) An appropriate threshold algorithm (image-adjust-auto threshold) and threshold results for all algorithms were selected. According to the results, the default algorithm was chosen here. 4) Parameters to be measured (analyze-set measurements) were set, and the mean gray value was checked and limited to threshold (very important), and OK was clicked. If the threshold limit was not checked, the average fluorescence intensity of the entire image was measured. 5) Measure was clicked, and the test result appeared. The measured results, mean, is the average fluorescence intensity (mean gray value, equal to IntDen/Area), IntDen = Integrated Density (sum of fluorescence intensity).

### ELISA

To measure IgA levels in colon tissues, feces and mucus were flushed and homogenized with endotoxin-free PBS. The supernatants were harvested after centrifugation (800G, 15 min) and stored at −80°C for later analysis. The cell supernatants of sort-purified B cells that underwent *in vitro* culture were also collected for cytokine and antibody measurements. The concentrations of IgA, IL-10, IL-6, and IgM were measured using the Mouse Ready-SET-Go! ELISA kits (eBioscience) according to the manufacturer’s instructions. The levels of active TGF-β were measured using a human TGF-β1 duo-set (DY240) or mouse TGF-β1 duo-set (DY1679) with a substrate reagent pack (DY999) according to the manufacturer’s instructions (R&D Systems Europe, Abingdon, UK) (25, 26).

### Statistical Analysis

The results of multiple groups were compared using a one-way analysis of variance (ANOVA). Thereafter, Bonferroni correction or the Mann-Whitney U test was conducted to compare two individual groups. Statistical analyses were performed using the GraphPad software (La Jolla, CA, USA). Statistical significance was set at P < 0.05.

## Results

### CD11b^+^ B Cells Are Enriched in the Intestine During DSS-Induced Colitis

**Figure 1:**
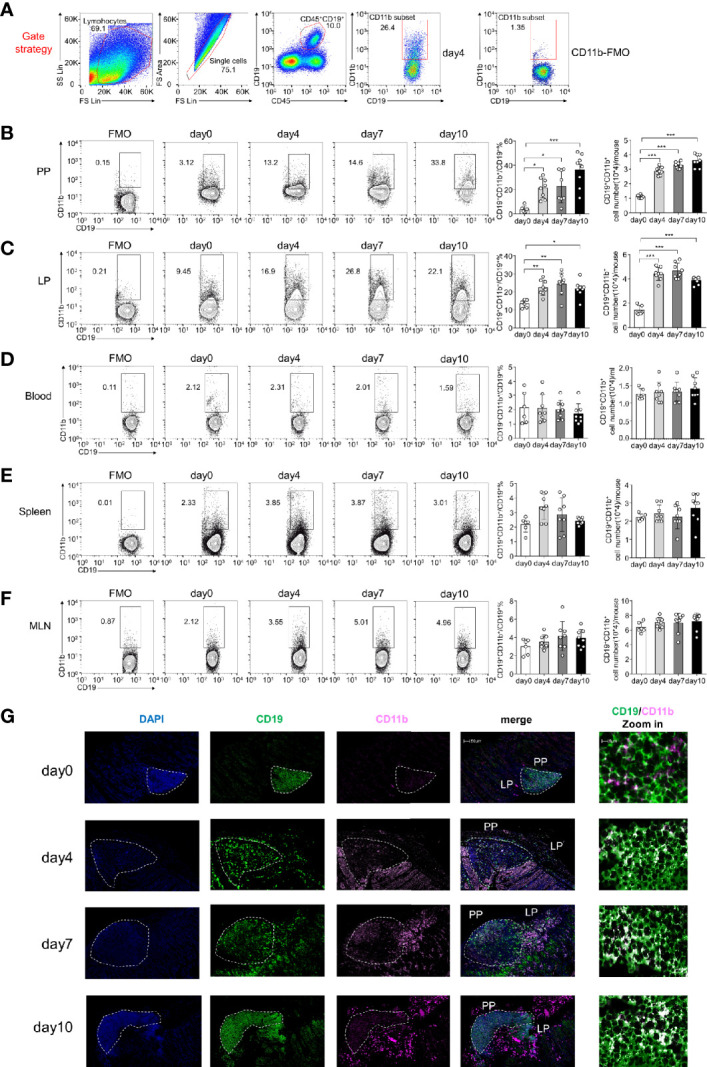
CD11b is induced in Peyer’s patches (PPs) and colorectal lamina propria (LP) B cells during dextran sulfate sodium (DSS)-induced colitis. Flow cytometry was used to analyze the frequency and absolute number of CD11b^+^ B cells. **(A)** Gating strategy for the identification of CD19^+^CD11b^+^ cells in gut-associated lymphoid tissue (GALT). Representative data from the LP of colitis mice on day 4 after DSS induction. Different fluorochrome-labeled isotype control antibodies or anti-CD45, -CD19, and -CD11b antibodies were used for staining. Numbers indicate the percentage of positive cells. The right panel represents CD11b fluorescence minus one (FMO) staining. Cells were isolated from the PPs **(B)**, colorectal LP **(C)**, total blood cell **(D)**, spleen **(E)**, and MLN **(F)** of DSS-induced WT mice on days 0, 4, 7, and 10. The absolute numbers were counting as follow: for PPs, colorectal LP, spleen, and MLN, the number of cells in that organ per mouse times the ratio of CD11b^+^B cells to obtain absolute count/mouse; for blood cell, 200ul blood cells were counted after red blood cell lysis, the number of 200ul blood cells times five and times the ratio of CD11b^+^B cells to obtain absolute count/ml. **(G)** Immunofluorescence of CD11b^+^CD19^+^ B cells in PPs and LPs. *P < 0.05, **P < 0.01, ***P < 0.001. Data are expressed as the mean ± SEM of one experiment with six to eight mice, performed in triplicate with similar results.

Our findings indicate that CD11b is upregulated on B cells during experimental autoimmune hepatitis (EAH) and plays an essential regulatory role in the disease (16). In this study, by using DSS-induced colitis, we detected B cells expressing CD11b at different stages in the PP and LP of GALT, peripheral blood, spleen, and MLN (**Figures 1A–F**). As shown in **Figures 1B, C**, during disease processing, the proportion of CD11b^+^ B cells increased on day 4 in the PP and LPs compared to that in day 0 control mice (day 0). Thereafter, they remained at high percentage levels and were accompanied by a significant increase in absolute cell numbers. However, there were no changes in CD11b^+^ B cell frequencies in the spleen or blood cells (**Figures 1D, E**). Moreover, our analysis also confirmed that the proportion of GALT CD11b^+^ B cells in total lymphocytes also increased (**Supplementary Figures S1A, B**), indicating that CD11b^+^ B cells were enriched in the GALT, specifically in the colitis stage. Immunofluorescence staining confirmed that CD11b^+^ B cells were mostly present in the intestinal PP and LP, especially under inflammatory conditions (**Figure 1G**).

Classically, CD11b is a hallmark of peritoneal cavity (PerC) B1 cells, which migrate to the intestine and maintain homeostasis (27, 28). Therefore, we compared intestinal (PP and LP) CD11b^+^ B cells with classic PerC B1 cells. We first compared the surface markers of PerC B1 cells and intestinal CD11b^+^ B cells before and after the induction of colitis. As shown in **Supplementary Figure S2A**, intestinal CD11b^+^ B cells exhibited higher levels of CD21, CD23, IgD, and IgA, as well as lower levels of IgM and CD5 on both day 0 and day 4, expressing an opposite phenotype from that of PerC B1 cells. To investigate whether the intestinal CD11b^+^ B cells are the PerC B1 B cells migrating to PP, PerC B1 B cells were isolated from CD45.1 mice and intraperitoneally injected into CD45.2 WT mice. An additional group of recipient mice was administered FTY720 to block the migration of injected PerC B1 B cells from the peritoneal cavity as a negative control (**Supplementary Figure S2B**). As shown in **Supplementary Figure S2C**, 4 days after cell transfer, peritoneal cells showed increased expression of S1P1. However, there was no migration of CD45.1 CD11b^+^ B cells to the PP or LP (**Supplementary Figure S2D**), strongly supporting that intestinal CD11b^+^B cells were not PerC B1 B cells. We then examined whether CD11b^+^B cells in LP and PP from day 0 to day 10 were the characteristic of memory B cells, plasmablasts, and germinal center (GC) B cells. We found that the frequency of both PP and LP CD11b^+^ B cells increased CD38^lo^PNA^hi^ (GC marker) expression on days 4, 7, and 10, while no changes were observed in the frequency of LP CD11b^-^ B cells (**Supplementary Figure S2E**). In addition, CD11b^+^ B cells expressed CD138 and Blimp from day 0 to day 10, and in inflammatory conditions, the frequency of CD138^+^Blimp^+^ plasmablasts increased in colitis (**Supplementary Figure S2F**). We also tested memory B cell markers, CD80 and PD-L2, and found that the levels of both markers were very low on CD11b^+^ B cells (**Supplementary Figure S2G**), indicating that CD11b^+^ B cells are not memory B cells. Taken together, these data suggest that CD11b^+^ B cells exist in all stages of B cell activation and could be expanded in PPs and colorectal LP during colitis and are highly unlikely to be conventional B-1 cells.

### Intestinal CD11b^+^ B Cells Protect Mouse From DSS-Induced Colitis

**Figure 2:**
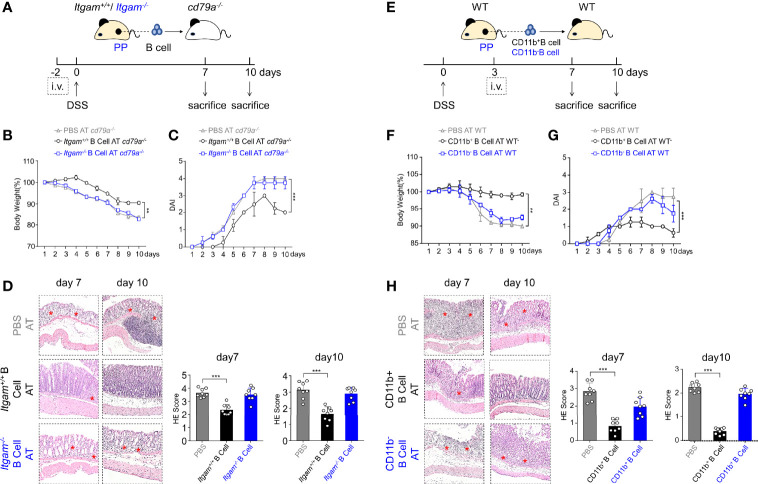
CD11b-deficient B cells exhibit impaired ability to resolve colitis. **(A)** Each *Cd79a^−/−^* mouse intravenously received 1 × 10^7^ PP-derived B cells from WT mice (*Itgam^+/+^*) or *Itgam^−/−^* mice 2 days before the induction of colitis. Mice were evaluated daily, and weight loss **(B)** and disease activity index (DAI) scores **(C)** were recorded. **(D)** Representative distal colon histological sections of *Cd79a^−/−^* mice were stained with hematoxylin and eosin (H&E), and the histological score was calculated. Images are displayed at the original magnification of ×100. Red stars indicate the position of the inflammatory cell infiltration and/or epithelial damage. **(E)** PP-derived B cells of WT mice were sorted and stimulated with lipopolysaccharide (LPS) for 48 h *in vitro*. CD11b^+^ and CD11b^−^ B cells were subsequently purified and intravenously injected (5 × 10^6^ cells per mice) into WT mice treated with DSS for 3 days. Weight loss **(F)** and the DAI scores **(G)** of recipient mice were measured and evaluated from day 0 to day 7. **(H)** H&E-stained histological sections of distal colon tissue are presented. Images are displayed at the original magnification of ×100. Red stars indicate the position of the histological injury. **P < 0.01, ***P < 0.001. Data are expressed as mean ± SEM of eight mice.

To determine whether CD11b^+^ B cells have a protective function in colitis, 1×10^7^ CD19^+^ B cells from the PP of day 0 WT (*Itgam^+/+^*) mice and CD11b gene-deficient (*Itgam^−/−^*) mice were purified, and AT was performed in B cell-deficient mice (*Cd79a^−/−^*) 2 days prior to the administration of DSS (**Figure 2A**). Compared to control mice (*Cd79a^−/−^* mice that received PBS), *Cd79a^−/−^* mice that received B cells from *Itgam^+/+^* mice exhibited less severe colitis. *Cd79a^−/−^* mice that received *Itgam^−/−^* B cells developed severe colitis similar to that in control mice, as measured by body weight loss, DAI score, and histological score (**Figures 2B–D**). *Cd79a^−/−^* mice that received *Itgam^+/+^* B cells lost <10% of their body weight on day 9 (the peak of disease progression) and did not experience diarrhea or gross bleeding after day 8. In contrast, *Cd79a^−/−^* mice that received *Itgam^−/−^* B cells exhibited persistent disease progression with nearly 20% body weight loss (**Figures 2B, C**). Histologically, control mice exhibited the most severe inflammation throughout the entire colon, with massive loss of crypts and leukocyte infiltration observed on days 7 and 10 (**Figure 2D**). *Cd79a^−/−^* mice that received *Itgam^−/−^* B cells displayed a slight improvement. Comparatively, *Cd79a^−/−^* mice that received WT B cells had an intact colon epithelium and markedly less leukocyte infiltration on days 7 and 10 (**Figure 2D**). These results indicate that the expression of CD11b in activated intestinal B cells is essential for their protective role in this colitis model. Further, we investigated whether intestinal CD11b^+^ B cells could be used to treat colitis. CD11b^+^ and CD11b^−^ B cells were sorted from PP, and an equal number of cells were added to WT mice 3 days after induction of DSS (**Figure 2E**). On day 7, mice that received CD11b^+^ B cells exhibited milder symptoms in the colon than mice that received CD11b^−^ B cells. Similar to mice that received PBS, mice that received CD11b^−^ B cells experienced body weight loss and diarrhea from day 3 (**Figure 2F**) and day 4 (**Figure 2G**), respectively. However, mice that received CD11b^+^ B cells exhibited delayed body weight loss and pathological changes related to colitis (**Figures 2F–H**). These data strongly suggest that intestinal CD11b^+^ B cells alleviate DSS-induced colitis and exhibit therapeutic effects.

### Intestinal CD11b^+^ B Cells Are the Main Source of IgA^+^ Cells

**Figure 3:**
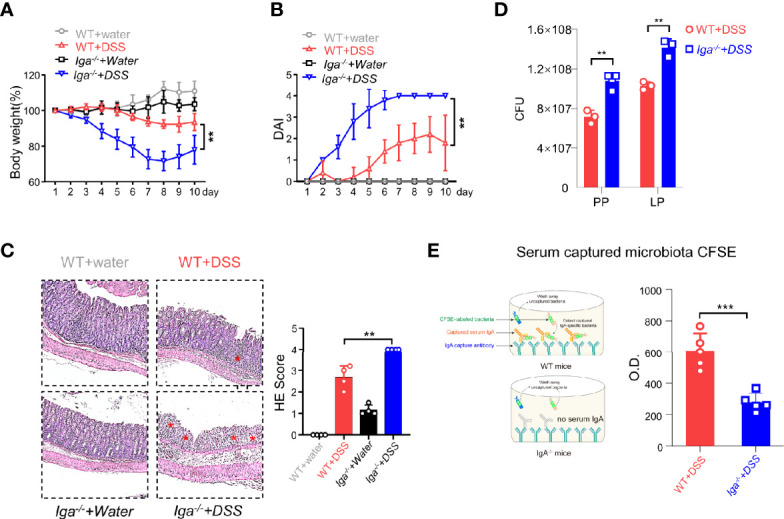
IgA in DSS-induced colitis. **(A)** *Iga^−/−^* mice and WT mice were administered DSS or regular water (water). Mice were evaluated daily, and weight loss and disease activity index (DAI) scores **(B)** were recorded. **(C)** Representative distal colon histological sections of *Iga^−/−^* mice were stained with hematoxylin and eosin (H&E), and the histological score was calculated. Images are presented at the original magnification of ×100. Red stars indicate the position of the inflammatory cell infiltration and/or epithelial damage. **(D)** The colony-forming units (CFU) counts of the bacteria in the feces of WT mice or *Iga^-/-^* mice on day 7 of colitis. **(E)** The O.D. values which indicated bacteria captured by the serum antibodies of WT mice or the *Iga^-/-^* mice on day 7 after DSS intervention were measured. **P < 0.01, ***P < 0.001. Data are expressed as mean ± SEM of three mice.

**Figure 4:**
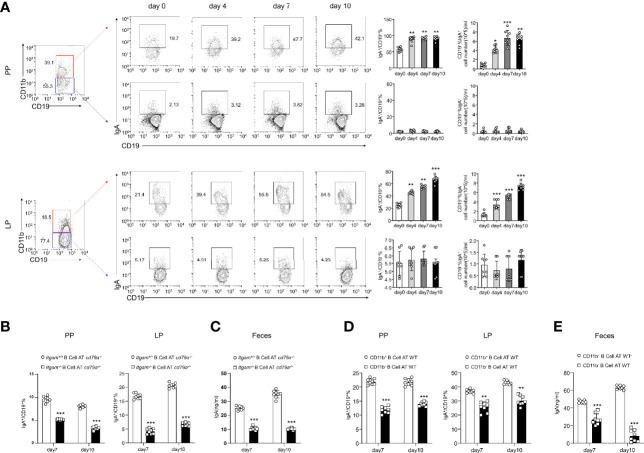
B cells expressing CD11b contribute to the differentiation of IgA in mice with colitis. **(A)** Expression levels of IgA in CD11b^+^ B cells and CD11b^−^ B cells in the PPs and colorectal LP at days 0, 4, 7, and 10 after treatment with DSS were detected using flow cytometry. The frequency and absolute number of IgA-expressing cells are presented. **(B)** The adoptively transferred *Itgam^+/+^* or *Itgam^−/−^* mice into *Cd79a^−/−^* mice were as described in **Figure 1**. The percentage and absolute number of IgA^+^ B cells in the LP and PPs from DSS-treated *Cd79a^−/−^* mice were analyzed using flow cytometry. **(C)** The supernatant of the mucus-containing fluid was harvested and transferred into 10 mL phosphate-buffered saline (PBS), and the production of sIgA in the colon was determined using enzyme-linked immunosorbent assay (ELISA). **(D)** The adoptively transferred CD11b^+^ and CD11b^−^ B cells into WT mice were as described in **Figure 1**. **(E)** The supernatant of the mucus-containing fluid was harvested and transferred into 10 mL PBS, and the production of sIgA in the colon was determined using ELISA. **P < 0.01; ***P < 0.001. Data are expressed as mean ± SEM of six mice.

IL-10 produced by B cells has been demonstrated to play an important role in the regulation of diseases (29). Thus, the role of IL-10 in CD11b^+^ B cells was investigated. As shown in **Supplementary Figures S3A-C**, the transfer of *Il-10^−/−^* CD11b^+^ B cells inhibited colitis, suggesting that IL-10 is not essential for CD11b^+^ B cell regulatory function. CD11b has been reported to affect the secretion of IgA in the lymphoid structure of PP (15). Herein, we used IgA-deficient (*Iga^−/−^*) mice to induce colitis and found that *Iga^−/−^* mice exhibited significantly more severe colitis symptoms than WT mice (**Figures 3A–C**). IgA-deficient (*Iga^−/−^*) mice developed more severe colitis than WT mice, as measured by body weight loss, DAI score, and histological score (**Figures 3A–C**). Consistently, *Iga^−/−^* mice had significantly higher numbers of bacteria in the PP and LP (**Figure 3D**), with lower bacterial-binding ability of antibodies in the blood (**Figure 3E**). Thus, IgA, a critical regulatory factor in maintaining gut homeostasis (30), was hypothesized to play a crucial role in CD11b^+^ B cell-mediated inhibition of colitis. As shown in **Figure 4**, CD11b^+^ B cells were gated to detect the expression of IgA in this subset, and this expression was compared to that measured in CD11b^−^ B cells in PPs and the LP. In contrast to CD11b^−^ B cells, CD11b^+^ B cells expressed more IgA during colitis, and the number of IgA^+^ cells was significantly increased in the colitis stage. However, increased numbers of IgA^+^ cells were not observed in CD11b^−^ B cells (**Figure 4A**). To confirm these results, we used *Iga^−/−^* mice as a negative control (**Supplementary Figure S4A**, PP day 10). Similar results were obtained from the transfer experiments with *Itgam^+/+^* B cells (**Figures 4B, C**) and isolated CD11b^+^ B cells (**Figures 4D, E**). Higher numbers of IgA^+^ B cells and levels of sIgA were observed in *Cd79a^−/−^* mice that received *Itgam^+/+^* B cells as well as WT mice that received CD11b^+^ B cells on days 7 and 10 after treatment with DSS, whereas *Itgam^−/−^* or CD11b^−^ B cell transfer did not increase the number of IgA^+^ B cells and sIgA expression (**Figures 4B–E**). In addition, the t-distributed stochastic neighbor embedding (tSNE) clustering visualization diagram also showed that IgA cells were present in CD11b^+^ B cells, indicating that the production of IgA required CD11b (**Supplementary Figure S4B**). Thus, CD11b^+^ B cells are the main source of IgA^+^ cells and represent the vast majority of IgA^+^ B cells.

### Activated TGF-β/p-Smad Signaling Is Mainly Activated in Intestinal CD11b^+^ B Cells

**Figure 5:**
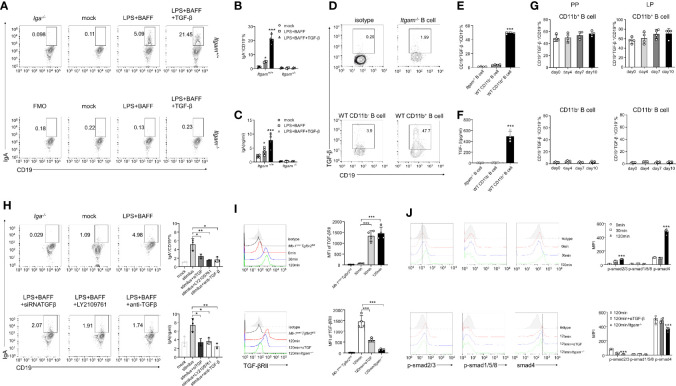
CD11b-deficient altering of Smad signaling in B cells resulted in reduced TGF-β production and IgA isotype switching. **(A)** Purified PP-derived *Itgam^+/+^* and *Itgam^−/−^* B cells of WT mice or Iga^-/-^ mice were stimulated with LPS, BAFF, and TGF-β for 72 h Subsequently, IgA^+^ B cells were analyzed using flow cytometry (The *Iga^-/-^* group was shown as a negative control). **(B)** The frequency of IgA-expressing cells is displayed. **(C)** The culture supernatant was harvested to determine the production of IgA *via* ELISA. **(D)** The population of TGF-β^+^ B cells in the PPs from DSS-treated mice was analyzed using flow cytometry. **(E)** The frequency of TGF-β-expressing cells is displayed. **(F)** Expression of TGF-β in mouse serum was measured using ELISA. **(G)** Expression of TGF-β in CD11b^+^ B cells and CD11b^−^ B cells in the PPs and colorectal LP at days 0, 4, 7, and 10 after treatment with DSS was detected using flow cytometry. The frequency and absolute number of IgA^+^ cells are presented. **(H)** The splenic B cells of mice were purified and transfected with small interfering RNA targeted for TGF-β (siTGF, other groups were transfected with the negative control siRNA for contrast). All groups were subsequently stimulated by LPS, BAFF, and TGF-β for 72 h *in vitro.* LY2109761 (5 µM) and anti-TGF-β (4 µg/mL) were used to inhibit TGF-β or TGF-β receptor, and the percentage of IgA^+^ B cells was measured using flow cytometry, and the IgA level in culture supernatant was measured using ELISA. The splenic B cells from the PP of WT mice, *Mb-1^cre/-^Tgfbr2^fl/fl^*, and *Itgam^−/−^* mice were purified and subsequently stimulated by LPS, TGF-β, or anti-TGF-β (αTGF, 4 µg/mL) for 0, 30, and 120 min. Expression of TGF-βRII **(I)** and the phosphorylation levels of Smad1/5/8 and Smad2/3 **(J)** were analyzed using flow cytometry. *P < 0.05; **P < 0.01; ***P < 0.001. Data are expressed as the mean of three independent experiments.

The mechanisms underlying the ability of CD11b^+^ B cells to differentiate into IgA^+^ cells and produce IgA were investigated. PP B cells derived from either *Itgam^+/+^* or *Itgam* ^−/−^ mice were cultured in the presence of LPS, BAFF, and TGF-β, all of which are required for class switching to IgA^+^ B cells. *Itgam^+/+^* B cells expressed IgA after stimulation compared to *Itgam* ^−/−^ B cells (**Figures 5A–C**), indicating that CD11b deficiency impaired the ability to produce IgA. However, the differentiation of IgM^+^ B cells and the production of sIgM were not affected (**Supplementary Figures S5A, B**). This indicates that CD11b deficiency selectively affected the class switching of IgA^+^ B cells but not the secretion of antibodies. Furthermore, the percentage of IgA^+^ cells and the level of sIgA among *Itgam^+/+^* B cells were significantly increased, even in the absence of TGF-β stimulation, compared with those of the unstimulated cells (**Figures 5A–C**). To investigate whether CD11b^+^ B cells could produce TGF-β themselves and thus trigger IgA production through an autocrine TGF-β-dependent loop, TGF-β production was measured in *Itgam* ^−/−^ PP B cells and WT CD11b^+^ PP B cells after stimulation with LPS. Almost none of the B cells derived from CD11b-deficient mice were TGF-β-positive, whereas >40% of TGF-β^+^ cells were detected among CD11b^+^ B cells derived from day-7 mice after the induction of colitis (**Figures 5D–F**). Furthermore, a significantly higher level of TGF-β was detected in intestinal CD11b^+^ B cells in day-7 mice with colitis (**Figure 5G**). In addition, **Figure 5G** shows increased TGF-β production in CD11b^+^ B cells. At the same time, there were no elevated frequencies of CD19^+^TGF-β^+^ B cells during colitis (compared to that in day 0). These findings demonstrate that CD11b^+^ B cells secrete TGF-β to promote the IgA class switch in response to intestinal inflammation in physiological and pathological states. To confirm these results, TGF-β siRNA(siTGF) and TGF-β/TGF-β receptor agonists were used to inhibit TGF-β/TGF-β receptor expression. Inhibition of the TGF-β/TGF-β receptor expression significantly decreased the expression of IgA in B cells (**Figure 5H** and **Supplementary Figure S5C**). Collectively, these results demonstrate that autocrine TGF-β is a possible mechanism involved in the enhanced ability of CD11b^+^ B cells to produce IgA.

The TGF-β-Smad signaling pathway has been implicated as a vital regulator of IgA class switching in activated B cells (31). Therefore, we sought to determine whether a deficiency in CD11b impaired the activation and function of the TGF-β-Smad pathway. PP-derived CD11b^+^ B cells were treated with LPS, BAFF, TGF-β, or anti-TGF-β (αTGF), and the expression levels of the TGF-β receptor and p-Smad were detected at 0, 30, and 120 min after stimulation. In WT CD11b^+^ B cells, the level of TGF-βRII expression was significantly increased at 30–120 min, p-Smad2/3 was phosphorylated at 120 min, and the expression of the co-factor Smad4 was increased at 120 min. However, the expression of Smad1/5/8 remained largely unchanged (**Figures 5I, J**). The expression of TGF-βRII, Smad4, and p-Smad2/3 was blocked when the anti-TGF-β antibody was used or B cells were obtained from *Itgam^−/−^* mice (**Figures 5I, J**). These results suggest that the expression of CD11b in B cells plays a positive role in regulating TGF-β signaling pathways, which are important for IgA class switching.

### CD11b^+^IgA^+^ B Cells Accumulate in the Human Intestine in UC and Are Associated With the Disease

**Figure 6:**
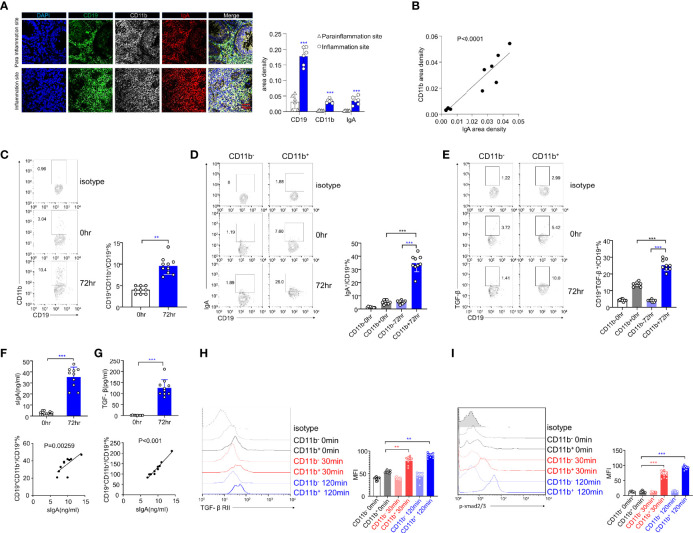
The expression levels of IgA and TGF-β/Smad pathway are upregulated in human CD11b^+^ B cells *in vitro*. **(A)** Representative staining of CD19 (green), CD11b (white), IgA (red), and 4′, 6-diamidino-2-phenylindole (DAPI) (blue) in the colon tissue of ulcerative colitis (UC) patients. The area density of each marker was calculated. Average data were collected from 20 random fields per patient, n = 6. **(B)** The relative area density of CD11b and IgA was identified. **(C)** Total PBMCs were purified and stimulated with CpG-ODN (2 μg/mL) and recombinant human IL-10 for 72 h *in vitro*. Cells were stained for CD11b detection and analyzed using flow cytometry. **(D)** The percentage of IgA^+^ B cells in CD11b^+^ and CD11b^−^ B cells was analyzed using flow cytometry. **(E)** The percentage of TGF-β^+^ B cells in CD11b^+^ and CD11b^−^ B cells was analyzed using flow cytometry. **(F)** The culture supernatant was harvested to determine the production of IgA (upper panel) *via* ELISA. The relative frequencies of CD19^+^CD11b^+^ cells and sIgA level were measured (lower panel). **(G)** The culture supernatant was harvested to determine the production of TGF-β (upper panel) *via* ELISA. The relative frequencies of CD19^+^CD11b^+^ cells and TGF-β level were measured (lower panel). The PBMCs from healthy donors were purified and subsequently stimulated by CpG and TGF-β for 0, 30, and 120 min. Expression of TGF-βRII **(H)** and the phosphorylation level of Smad2/3 **(I)** were analyzed using flow cytometry. **P < 0.01; ***P < 0.001. Data represent the mean of more than three independent experiments.

We obtained intestinal sections from six UC patients and stained them with DAPI and CD19, CD11b, and IgA antibodies for immunofluorescence analysis. The inflammatory site was found to be heavily infiltrated with CD19^+^CD11b^+^IgA^+^ cells (**Figure 6A**, upper panel), and the area densities of CD19, CD11b, and IgA were significantly higher at the inflammation site (**Figure 6A**, right panel). In addition, the area density of CD11b was positively correlated with that of IgA (**Figure 6B**). These results indicate that CD11b^+^IgA^+^ B cells exist in the intestinal tract of UC patients to maintain homeostasis. Next, we used CCR9 and α4β7 to screen for intestinal circulation-derived cells, sorted cells with high expression levels of CCR9 and α4β7 from the peripheral blood of healthy donors and induced CD11b and IgA expression *in vitro* (**Supplementary Figure S6**). CD11b expression was upregulated in human B cells at 72 h after CpG stimulation (**Figure 6C**). In addition, IgA and TGF-β expression was upregulated in CD11b^+^ B cells after 72 h of stimulation compared to that in CD11b^-^ B cells (**Figures 6D, E**). Furthermore, the number of IgA^+^ B cells and sIgA levels increased after 72 h of stimulation (**Figure 6F**). Notably, CD11b^+^ B cells expressed more IgA than CD11b^−^ B cells, and the percentage of CD19^+^CD11b^+^ B cells was positively correlated with sIgA levels (**Figure 6F**). These data suggest that, similar to murine cells, CD11b and IgA expression was upregulated in B cells after stimulation. Furthermore, the TGF-β/TGF-βR/p-Smad signaling pathway was also investigated. As shown in **Figures 6E, G**, a significantly higher level of TGF-β in cultured CD11b^+^ B cells was detected *in vitro* than that in CD11b^−^ B cells. Moreover, the percentage of CD19^+^CD11b^+^ B cells was positively correlated with the level of secreted TGF-β. The expression levels of TGF-βRII and p-Smad2/3 were significantly increased in CD11b^+^ B cells at 30 min and 120 min (**Figures 6H, I**), indicating that the expression of CD11b in human B cells plays a positive role in regulating TGF-β signaling pathways. Altogether, we demonstrated that GALT CD11b^+^ B cells proliferated under inflammatory conditions, inhibiting intestinal inflammation by secreting sIgA through the TGF-β/p-Smad2/3 signaling axis (**Supplementary Figure S7**).

## Discussion

CD11b and other members of the β2 integrin family are usually expressed on myeloid cells and play important roles in provoking or suppressing the inflammatory effects by regulating immune cell migration, interaction, and signal transduction (32). In this study, the DSS-induced colitis model was used to further explore the multiple functions of CD11b^+^ B cells in diseases. To further investigate these functions, the increase in the number of CD11b^+^ B cells in the blood, spleen, and GALT during colitis was first documented. CD11b^+^ on B cells in the LP and PP was highly expressed, consistent with the results of previous studies (33, 34). Furthermore, there was no significant increase in the number of CD11b^+^ B cells in peripheral immune organs, indicating that CD11b^+^ B cells were likely cells originating from the intestine in colitis. Many CD11b^+^ B cells were also recognized in the PP and LP on day 0, possibly because the GALT was constantly in contact with microbiota and foods and was always in a state of physiological inflammation; thus, CD11b^+^ B cells were required to maintain homeostasis.

Traditionally, CD11b is expressed on mouse B-1 cells, which are a small population of B cells preferentially located in the peritoneal cavity and pleural cavity and are rare in lymph nodes (35, 36). The majority of B1 cells have relatively constant phenotypes (CD5 or CD11b) and are normally produced during fetal/neonatal development (37). It has been reported that B1-b B cells can migrate into the colorectal LP and switch into IgA^+^ B cells (38, 39). To compare CD11b^+^ B cells and B-1 B cells, CD19^+^ cells from the peritoneal cavity, colorectal LP, and PPs of WT mice were purified by FACS and sorted into CD5^+^ CD11b^+^ (B-1a), CD5^-^ CD11b^+^/CD11b^hi^ (B-1b), and CD5^+^ CD11b^-^ (B-1c) cells. After colitis induction, we were able to detect significantly increased numbers of CD5^-^ CD11b^+^ B cells, which showed marked proliferation (Ki67^+^ cells) in colorectal LP and PPs but not in the peritoneal cavity (data not shown). This distinction is important because B1 B cells do not actively proliferate, whereas B2 B cells have the ability to vigorously proliferate in GCs (40). In addition, the surface markers of induced CD11b^+^ B cells and B1 cells differed significantly. In particular, the PP CD11b^+^ B cells had exactly the same phenotype as B-2 cells with high expression of CD23 and low expression of CD5/IgM, while colorectal LP CD11b^+^ B cells are still under debate. It is not clear whether the induced GALT CD11b^+^ B cells are devoid of B1 cells, but it is highly probable that GALT CD11b^+^ B cells are B-2 cells that can be induced in response to the inflammatory microenvironment and constitute a new fraction of the intestinal B cell subset. At the same time, we also did BCR diversity analysis to confirm our conclusion (data not shown). We aimed to identify different markers to distinguish CD11b^+^ B cells from B1 B cells. As we believe that GALT CD11b^+^ B cells tend to belong to B-2 cells, the stage of B-2 cells needs to be clarified. We detected PP and LP B cells from day 0 to day 10 and divided them into the GC phase to reveal the process and memory B cells or plasmablasts to reveal the outcome. It was found that CD11b^+^ cells belonged to GC B cells and plasmablasts, which was consistent with their high proliferation characteristics demonstrated previously and also consistent with the results of our previous study (16). Considering that the mucosal immune system was in a state of chronic inflammation, PPs, as the main site of B cell differentiation, were in a state of activation for the delivery of IgA. Thus, our data showed that PP CD11b^+^ cells were CD38^+^PNA^+^ and CD138^+^Blimp^+^ on day 0. Microorganisms occupy nearly 50% of the body, and the presence and imbalance of intestinal flora are the key factors inducing many diseases, especially colitis. They have a direct or indirect impact on the initial differentiation of B cells. The components of bacteria, such as LPS and flagellin, are ubiquitous in the intestinal microenvironment. We found that CD11b was highly expressed to some extent after LPS stimulation, but there was no such change after anti-BCR stimulation (data not shown). TLRs on the surface of B cells are involved in the differentiation process of B cells in GC and induce the expression of AID, and it has been reported that LPS mediates long-term primary class-switched antibody responses and memory-like antibody responses *in vivo* (41, 42).

The AT system was subsequently used to verify the role of CD11b^+^ B cells, demonstrating that *Itgam^−/−^* B cells could not resolve colitis. To exclude the influence of microbiota, mice from different groups were housed for over one month. As the results were consistent with those of previous studies, CD11b is further indicated to be indispensable for the suppressive role of B cells in immune responses (43, 44). Currently, there are numerous therapeutic strategies for inflammatory bowel disease (IBD) patients. Novel agents that target proinflammatory cytokines (i.e., tumor necrosis factor α, IL-12, and IL-23) are currently undergoing clinical investigation (45). However, these anti-cytokine treatments appear to be effective only in certain subgroups of patients (46, 47). Thus, LPS-induced CD11b^+^ B cells have been used as therapeutic agents against colitis. AT of these CD11b^+^ B cells in mice was performed during the early stage of DSS treatment, following the manifestation of symptoms (e.g., diarrhea). Previously, we demonstrated that CD11b^−^ B cells can be induced to form CD11b^+^ B cells; thus, they also possess a certain ability to alleviate the disease. However, in the present study, CD11b^−^ cells were isolated after 48 h of *in vitro* culture. As a result, their ability to transform into CD11b^+^ B cells was limited, thereby negatively affecting their ability to alleviate the disease. In conclusion, this transferred cell-based therapy in a colitis mouse model may be a potential clinical therapeutic approach for Crohn’s disease and UC patients.

Growing evidence indicates that IgA plays an important role in preventing potentially invasive commensal bacteria and neutralizing microbial toxins in the gut (48, 49). IgA deficiency in humans has been associated with chronic intestinal inflammatory disorders (50). However, the exact subset of B cells that possess this IgA-related suppressive function remains debatable. Macpherson et al. reported that a large amount of IgA in the intestinal LP is derived from peritoneal B cells in mice (51), while Thurnheer et al. demonstrated that the upregulation of intestinal IgA level induced by commensal bacteria is mainly mediated by conventional B2 cells and not B1 cells (52). Kunisawa et al. demonstrated that the function of CD11b^+^ B cells is related to the classic mucosal antibody IgA. They reported that CD11b^+^IgA^+^ plasma cells, unlike CD11b^−^IgA^+^ cells, are the major source of IgA, and the authors did not elaborate on the mechanism (15). In our study, the expression of IgA correlated with the expression of CD11b in B cells in the LP and PPs of mice with colitis. Naïve B cells were also found to be initially induced to form CD11b^+^ B cells. Thereafter, CD11b^+^ B cells were differentiated into IgA^+^ plasmablasts, referred to as CD11b^+^IgA^+^ plasmablasts, in both the PPs and LP. However, CD11b^−^ and *Itgam^−/−^* B cells did not possess this property of IgA class switching. In the *in vitro* assay performed in this study, phosphorylation of Smad2/3 was not observed in *Itgam^−/−^* B cells derived from PPs, whereas WT CD11b^+^ B cells demonstrated activated p-Smad2/3 after stimulation. These findings suggest that CD11b may promote the activation of Smad proteins and consequently facilitate the production of IgA. We also found that B cells secrete TGF-β and complete IgA production autocrine. However, TGF-β activation is a multiple-step process, including the release of TGF-β from TGF-β/LAP complexes (25). We did not provide evidence of the activation of TGF-β in B cells, which we may explore in the next project.

In summary, the findings of the present study reveal that intestinal CD11b^+^ B cells ameliorate DSS-induced colitis and maintain gut homeostasis. Based on *in vitro* and *in vivo* experiments, CD11b^+^ B cells exhibiting high activation of the TGF-β-Smad signaling pathway were the primary source of IgA, which maintained gut homeostasis and mitigated colitis. These phenomena were also confirmed in UC patients and *in vitro* experiments using human cell cultures, indicating the clinical application value of this study.

## Data Availability Statement

The original contributions presented in the study are included in the article/**Supplementary Material**, further inquiries can be directed to the corresponding authors.

## Ethics Statement

The studies involving human participants were reviewed and approved by the medical ethics council of Zhongshan Hospital. The patients/participants provided their written informed consent to participate in this study. The animal study was reviewed and approved by animal facility of Fudan University. Written informed consent was obtained from the owners for the participation of their animals in this study.

## Author Contributions

YF, ZW and LW designed and performed the experiments, analyzed data, and wrote the manuscript. CY designed the experiments and wrote the manuscript. ML, WX, YLin, and EH improved the manuscript. BY, YLiu, RL, and HL performed the experiments and analyzed the data. The first two and the last two authors contributed equally to this work. All authors contributed to the article and approved the submitted version.

## Funding

This work was supported by the General Program of the National Natural Science Foundation of China (81771736, 81971493, 31770992), Major Program of National Natural Science Foundation of China (81730045), Shanghai Rising-Star Program (20QA1407900), and Innovative Research Team of High-level Local Universities in Shanghai.

## Conflict of Interest

The authors declare that the research was conducted in the absence of any commercial or financial relationships that could be construed as a potential conflict of interest.

## Publisher’s Note

All claims expressed in this article are solely those of the authors and do not necessarily represent those of their affiliated organizations, or those of the publisher, the editors and the reviewers. Any product that may be evaluated in this article, or claim that may be made by its manufacturer, is not guaranteed or endorsed by the publisher.

## Abbreviations

APC, allophycocyanin; AT, adoptive transfer; BAFF,B-cell-activating factor of the tumor-necrosis-factor family; CCR, CC chemokine receptor; CFSE, carboxyfluorescein succinimidyl ester; CFU, colony-forming units; DAI, disease activity index; DAPI, 4’, 6-diamidino-2-phenylindole; DSS, dextran sodium sulfate; FITC, fluorescein isothiocyanate; GALT, gut associated lymphoid tissues; IBD, inflammatory bowel disease; IgA, Immunoglobulin A; LP, lamina propria; LPS, lipopolysaccharide; MLN, mesenteric lymph nodes; p-Smad, phosphorylated Smad; PBS, phosphate-buffered saline; PE, phycoerythrin; PP, peyer’s patch; rm, recombinant mouse; TGF-β, cytokine transforming growth factor β.
