# Supplementary material for: Intestinal CD11b+ B Cells Ameliorate Colitis by Secreting Immunoglobulin A
Source: Front Immunol. 2021 Nov 3;12:697725. doi: 10.3389/fimmu.2021.697725 (PMC8595478; doi:10.3389/fimmu.2021.697725)
Supplement: Supplementary file 1 [file DataSheet_1.pdf]

Supplemental Information

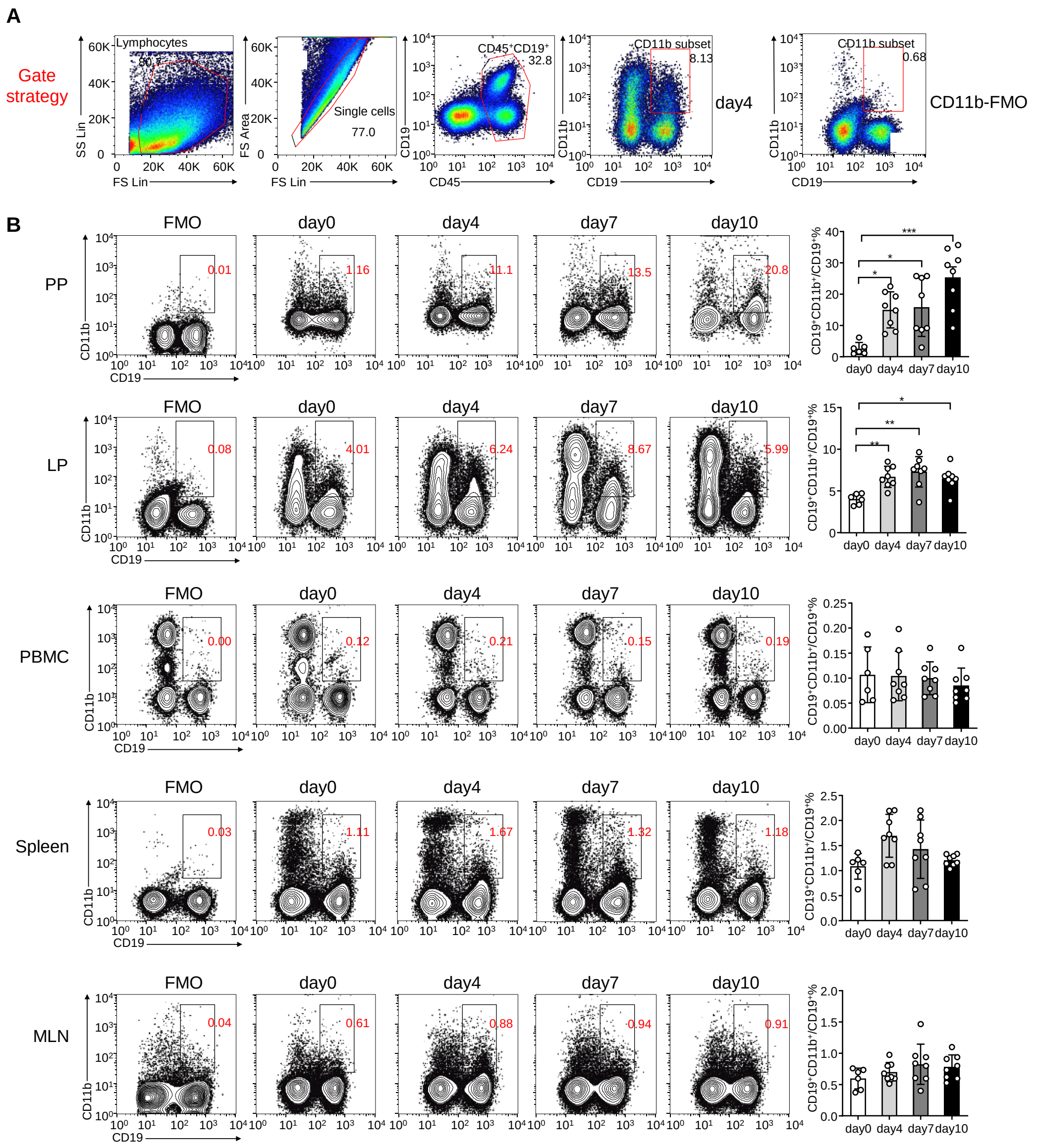

Figure S1. CD11b is induced in Peyer's patches and colorectal LP lymphocytes during DSS-induced colitis.

Flow cytometry was used to analyze the frequency of CD11b expression in CD45$^+$ cells. (**A**)**.** Gating strategy for the identification of CD45$^+$ cells in GALT. Representative data from LP of colitis mouse on Day 4 after DSS induced stained with different fluorochrome-labeled isotype control antibodies or anti-CD45, CD19, and CD11b antibodies. Numbers indicate the percentage of positive cells. Right panel is the CD11b fluorescence minus one (FMO) staining. **(B).** Cells were isolated from the Peyer's patches (PP), the colorectal lamina propria (LP), peripheral Blood Mononuclear Cell

(PBMC) , spleen, and the mesenteric lymph node (MLN) of DSS-induced WT mice on days 0, 4, 7, and 10. **P < 0.01; ***P < 0.001. Data shown are the mean ± SEM from one experiment with six to eight mice, performed in triplicate with similar results.

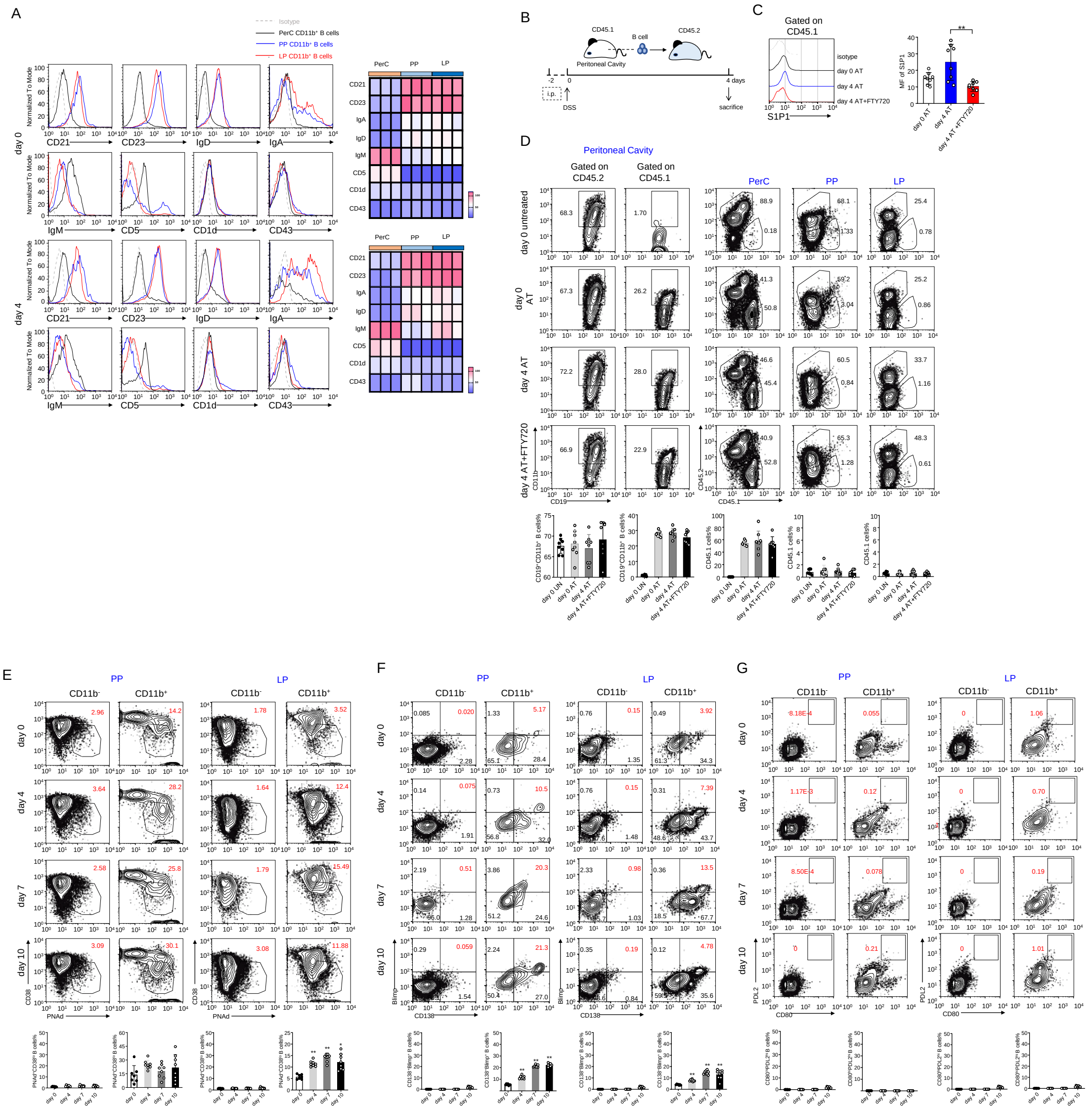

Figure S2. Expression profile in intestinal B cells during DSS-induced colitis.

(A). Differential markers on CD11b$^+$ B cell gated in PerC, PPs, and LP were detected through flow cytometry (left panel). Heat maps show the profiles of surface marker (right panel). **(B).** PerC B1 B cells from CD45.1 mice were harvested and intraperitoneally injected into CD45.2 WT mice. Subsequently, the CD45.2 WT mice were treated with DSS for 4 days. The mice were divided into PBS-injected (day 4), CD45.1 B cell-transferred (day 4 AT), and CD45.1 B cell-transferred with

intraperitoneal injection of FTY720 (day 4 AT + FTY720) groups. The FTY720 (1mg/kg) was intraperitoneal injected every day until sacrificed. **(C).** The expression of S1P1 in PerC cells was detected through flow cytometry. **(D).** The CD45.1 or CD45.2 $CD11b^+$ B cells of PerC, PP, or LP were detected through flow cytometry. The surface markers of $CD11b^-$B cells and $CD11b^+$ B cells in LP and PP from day 0 to day 10 after DSS induction were detected by flow cytometry to verify the germinal center B cells **(E)**, plasmablast**(F)** and memory B cells**(G)**markers. **P < 0.01. Data shown are the mean ± SEM of seven mice or more.

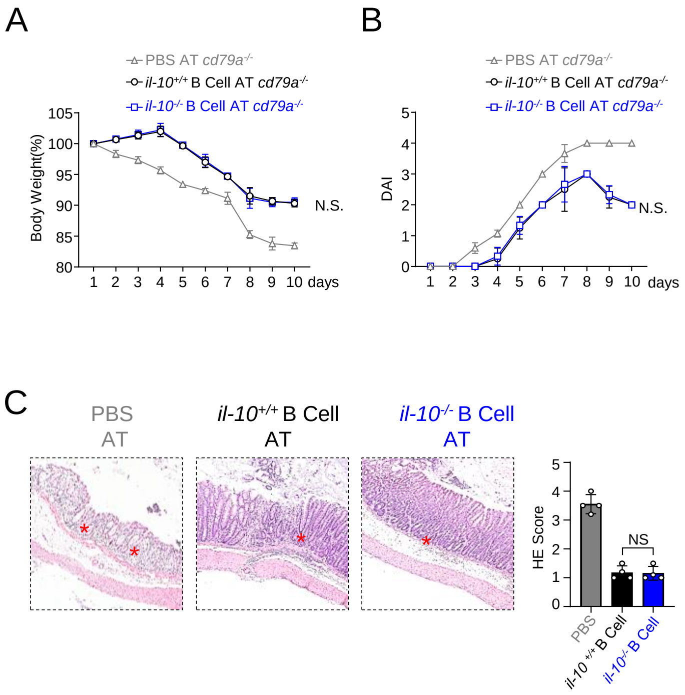

Figure S3. Adoptively transferring of *il-10*$^{-/-}$ mice $CD11b^+$ B cells

PP-derived $CD11b^+$B cells of WT mice or *Il-10*$^{-/-}$ mice were sorted and intravenously injected (2×10^7 cells per mice) into *Cd79a*$^{-/-}$ mouse 2 days before of the DSS induction. Weight loss **(A)** and the DAI scores. **(B)** of the recipient mice were measured and evaluated from day 0 to day 10. **(C).** Colon tissues from the recipient mice on days 7 after the induction of colitis, H&E-stained histological sections of distal colon tissue is shown. Images are shown at original magnification ×100. A red asterisk indicates the position of the histological injury.

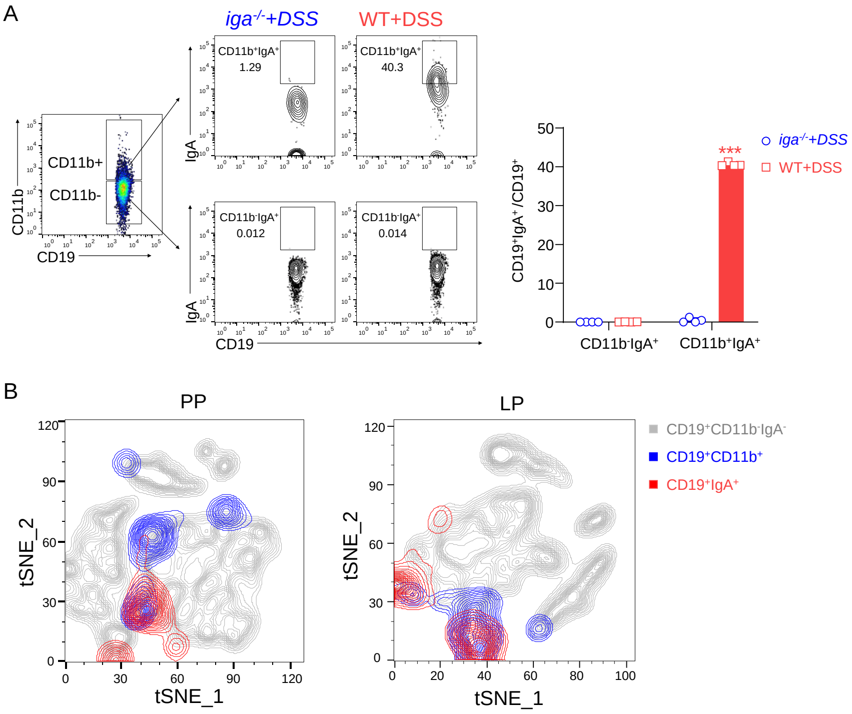

Figure S4. IgA^{+} cells was observed in CD11b^{+} B cells

(A). The expression level of IgA in CD11b^{+} B cells and CD11b^{-} B cells in PPs from DSS-treated *Iga*$^{-/-}$ mice and WT mice. **(B)**. The t-Distributed Stochastic Neighbor Embedding (tSNE) clustering visualization of B cells of PPs and LPs from DSS-treated WT mice at day7. ***P < 0.001. Data are expressed as mean ± SEM of four mice.

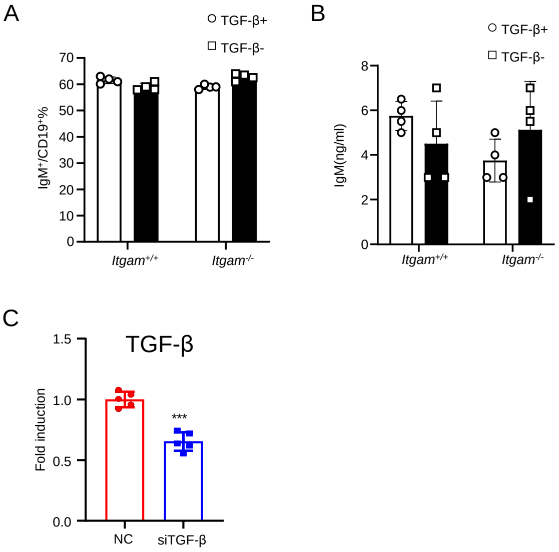

Figure S5. The expression of IgM in *Itgam*$^{+/+}$ and *Itgam*$^{-/-}$ B cells using an in vitro assay.

(A). Purified PPs-derived *Itgam*$^{+/+}$ and *Itgam*$^{-/-}$ B cells were stimulated with or without TGF-β for 72 h. IgM^{+} cells were detected using flow cytometry. **(B).** The production of sIgM was detected by ELISA. **(C).** The mRNA expression of TGF-β at 72h after siRNA transfection with the LPS and BAFF stimulation. ***P < 0.001. Data are expressed as mean ± SEM of five mice.

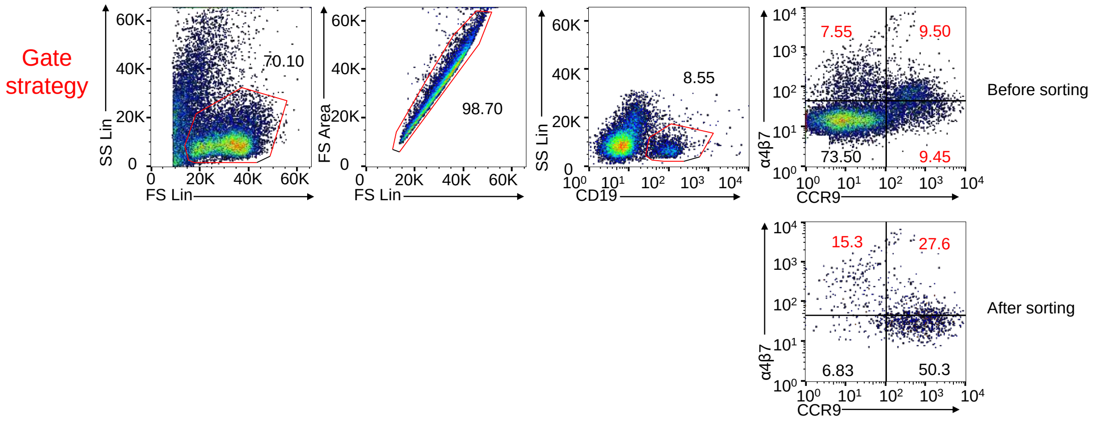

Figure S6. FACS purified gut specific B cells

PBMC CCR9 and/or α4β7 were FACS purified and representative dot plots before and after sorting are shown. Cells were stained combinations of anti-CD19, CCR9 and α4β7 or their isotype-matched controls.

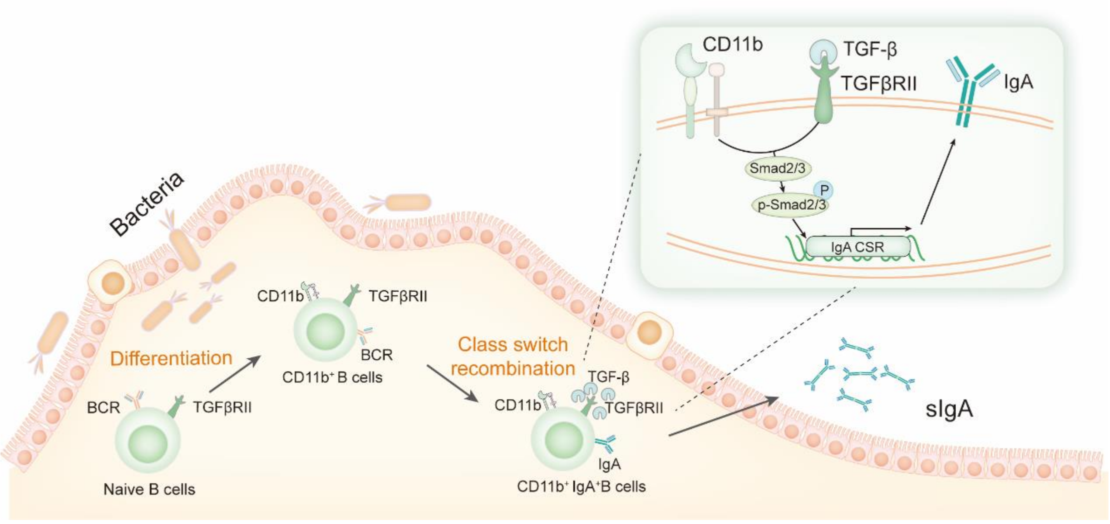

Figure S7. A schematic view of the GALT CD11b^{+} B cell differentiation and function.
